# Establishment and Validation of a Transdermal Drug Delivery System for the Anti-Depressant Drug Citalopram Hydrobromide

**DOI:** 10.3390/molecules29040767

**Published:** 2024-02-07

**Authors:** Yi-yang Sun, Ya-jing Ni, Run-jia Wang, Zi-cheng Qin, Zhao Liu, Li-hui Xiao, Yan-qiang Liu

**Affiliations:** 1 College of Life Sciences, Nankai University, Tianjin 300071, China; yiyangsunsun@163.com (Y.-y.S.); n2870239057@163.com (Y.-j.N.); wangrunjia202205@163.com (R.-j.W.); qinzichen2021@163.com (Z.-c.Q.); 2 Harvest Pharmaceutical Co., Ltd., Changsha 410000, China; liuz@harvest-pharm.com (Z.L.); xiaolh@harvest-pharm.com (L.-h.X.)

**Keywords:** transdermal delivery, citalopram hydrobromide, pharmacokinetics, skin irritation

## Abstract

To enhance the bioavailability and antihypertensive effect of the anti-depressant drug citalopram hydrobromide (CTH) we developed a sustained-release transdermal delivery system containing CTH. A transdermal diffusion meter was first used to determine the optimal formulation of the CTH transdermal drug delivery system (TDDS). Then, based on the determined formulation, a sustained-release patch was prepared; its physical characteristics, including quality, stickiness, and appearance, were evaluated, and its pharmacokinetics and irritation to the skin were evaluated by applying it to rabbits and rats. The optimal formulation of the CTH TDDS was 49.2% hydroxypropyl methyl cellulose K_100M_, 32.8% polyvinylpyrrolidone K30, 16% oleic acid-azone, and 2% polyacrylic acid resin II. The system continuously released an effective dose of CTH for 24 h and significantly enhanced its bioavailability, with a higher area under the curve, good stability, and no skin irritation. The developed CTH TDDS possessed a sustained-release effect and good characteristics and pharmacokinetics; therefore, it has the potential for clinical application as an antidepressant.

## 1. Introduction

Major depressive disorder is a neuropsychiatric disease that affects more than 350 million people worldwide [1]. It is a disorder of affect or mood caused by various factors and is also known as depressive disorder. Its main features include anxiety, anhedonia, low mood, and attention deficit. Most patients also have somatic symptoms, and severe cases can lead to suicide [2,3,4]. Depression is different from a transient depressive mood and is a lifelong disease that may recur. Most patients with first-onset depression are approximately 30 years of age, and it has a lifetime risk of 15–18%. Women are more affected by depression than men [5,6]. Clinical research results have shown that depression has high incidence and recurrence rates, but a low cure rate. It seriously damages people’s physical and mental health, affects their personal and work life, and imposes major economic and mental burdens on families and society [7].

Commonly used antidepressants in clinical practice are monoamine oxidase inhibitors, tricyclic antidepressants, selective serotonin (5-HT) reuptake inhibitors, 5-HT and norepinephrine (NE) reuptake inhibitors, and NE and selective 5-HT reuptake inhibitors [8,9]. Among these, selective 5-HT reuptake inhibitors have been shown to be reliable, safe, and well-tolerated drugs; therefore, they have been increasingly used as the first-choice antidepressant in clinical practice [10,11,12].

Citalopram hydrobromide is marketed as Cipramil, and its active ingredient is citalopram [13,14,15], which is a selective serotonin reuptake inhibitor. Some studies have shown that the oral medication compliance of patients with depression is low, at only 40–70% [16], and oral medication cannot achieve good treatment effects. The oral administration of citalopram hydrobromide (CTH), a commonly used antidepressant, can cause gastrointestinal irritation, such as nausea, vomiting, diarrhea, constipation, appetite change, and dry mouth, affecting patient compliance and quality of life [17]. The incidence and severity of these side effects are related to the type of drug, the dose, duration of use, mode of administration, and individual patient differences [18]. Reducing the side effects and improving the medication compliance of patients to achieve good therapeutic effects and wider clinical applications of CTH have become key problems to be solved urgently [19].

A transdermal drug delivery system (TDDS) delivers drugs through the skin, providing local or systemic drug effects, mainly through diffusion [20,21]. TDDSs have the advantages of avoiding interference and degradation of the gastrointestinal tract and liver, improving the bioavailability and therapeutic effect of drugs, reducing side effects and discomfort, facilitating use in children and older adults, realizing local and systemic treatment, controlling the time of drug release, and having the ability to stop drug administration at any time [22,23,24]. The first transdermal patch for the treatment of depression was the Selegiline Transdermal System, which was marketed in 2006 [25,26,27]. Since the introduction of this patch, the development of transdermal antidepressant drug delivery systems has attracted considerable attention. Paroxetine is a selective serotonin reuptake inhibitor used to treat mild-to-moderate depressive symptoms. Pathan et al. used soy lecithin and ethanol to prepare ethanol bodies as carriers for the percutaneous delivery of paroxetine hydrochloride, and they established a potentially safe percutaneous delivery system for paroxetine hydrochloride [28]. The drug was approved for marketing by the U.S. Food and Drug Administration in May 2019. Fluoxetine is a commonly used antidepressant. Singh et al. developed a matrix fluoxetine transdermal patch via solvent volatilization using a polymer combination of EC and PVP [29]. However, there have been few studies on transdermal drug delivery systems for CTH. So, in this study, to establish a transdermal delivery system for CTH, an in vitro skin release experiment applying mouse abdominal skin (mouse skin has high permeability and is suitable for the repeatability and comparability of in vitro trans-dermal experiments) was performed to screen for the best formulation components and proportions (to evaluate the performance of these systems, in vitro methods are used to simulate the skin conditions and drug transport mechanisms) applying the Franz diffusion cell [30,31], followed by a pharmacokinetic study and skin irritation evaluation applying rabbits (rabbit skin is similar to human skin in structure and function, and has a large skin area, which facilitates blood sampling and pharmacokinetic analysis). We aim to provide a new delivery method for CTH to make CTH more effective against depression. This study will compensate for the shortcomings in this field and provide a reference for developing transdermal patches of CTH.

## 2. Results

### 2.1. Preselection of the Transdermal System

The results of the preliminary screening of the patch formulations are shown in Table 1. We found that the final cumulative percentage of CTH depended on the different components of the system. When the primary excipient material was HPMCK_100M_, which has a high molecular weight, the secondary excipient material was PVA, and the permeability promoting agent was OA:azone (1:1), the CTH TDDS showed the best release and transdermal performance, with a cumulative release rate reaching 40.82% within 24 h. This was markedly higher than the release rates of the other combinations. Therefore, our preliminary matrix formulation was based on HPMCK_100M_ as the primary excipient material, PVA as the secondary excipient material, and OA:azone at a ratio of 1:1 as the penetration enhancer.

### 2.2. Determination of the Optimal Composition of Excipient Materials in the Transdermal System

**Figure 1:**
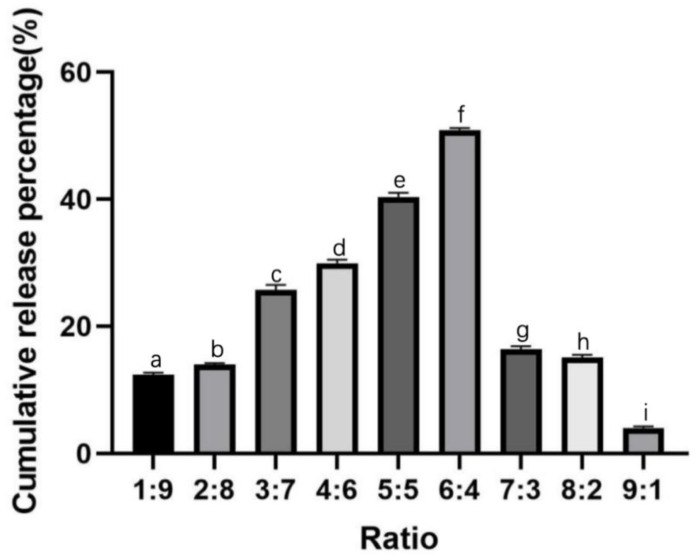
Effect of the excipient material ratio on the in vitro release of CTH from the transdermal drug delivery system. Data are presented as the mean ± standard deviation (SD; n = 3). The marked different letters indicate significant difference among treatments. CTH, citalopram hydrobromide.

With an increase in HPMCK_100M_ mass fraction (A1–A9; Table 2), the cumulative release rate of CTH in vitro showed a trend of first increasing and then decreasing. When the mass ratio of HPMCK_100M_ and PVA was 6:4, the CTH transdermal delivery system had the highest release effect, and the cumulative release rate reached 50.88 ± 0.29% within 24 h, which exceeded the release rate for the other ratios (Figure 1). Therefore, this ratio of the excipient materials was used in the transdermal system.

### 2.3. Determination of the Optimal Penetration Enhancer Content of the Transdermal System

**Figure 2:**
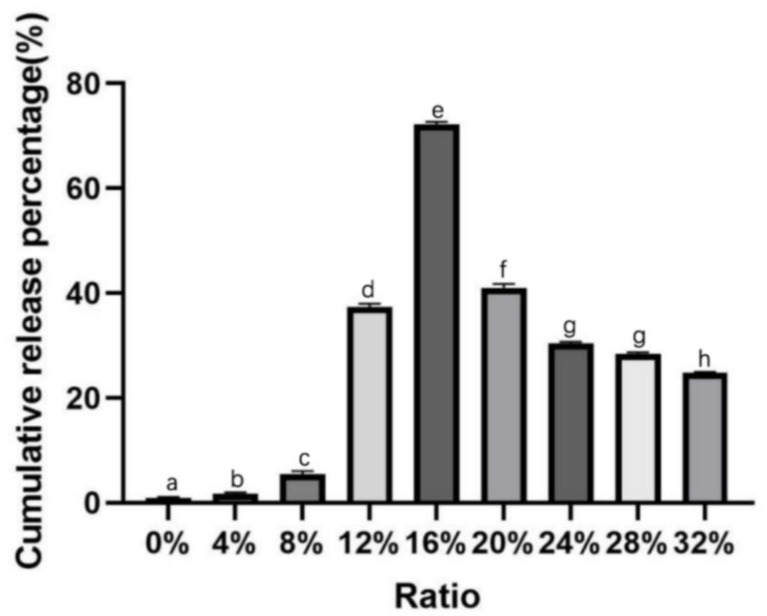
Effect of the penetration enhancer concentration on the in vitro release of CTH from the transdermal drug delivery system. Data are presented as the mean ± SD (n = 3). The marked different letters indicate significant difference between treatments, and the same letters indicate no significant difference between treatments.

Nine components (B1–B9; Table 2) were selected to determine the optimal penetration promoter for the transdermal system. Figure 2 shows the effects of different concentrations of OA/azone mixtures on osmotically promoted CTH release. The release rate of CTH in vitro first increased and then decreased with increasing osmotic agent concentration. In addition, when the mass ratio of OA/azone (1:1) was 16%, the CTH transdermal delivery system had the highest release effect, with a cumulative release rate of 72.25 ± 0.47% within 24 h, which was significantly higher than the release rates for other mass fraction combinations. Therefore, B5, which comprised 16% OA/azone, was selected for further use.

### 2.4. Determination of the Optimal Content of Polyacrylic Acid Resin II in the Transdermal Systems

**Figure 3:**
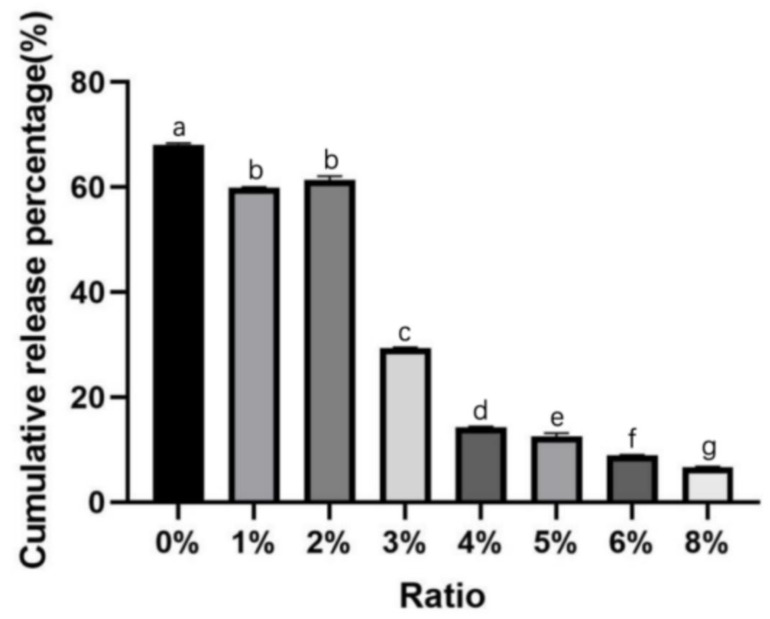
Effect of polyacrylic acid resin II on the in vitro release of CTH from the transdermal drug delivery system. Data are presented as the mean ± SD (n = 3). The marked different letters indicate significant difference between treatments, and the same letters indicate no significant difference between treatments.

To investigate the optimal content of the pressure-sensitive adhesive (PSA) polyacrylic resin II in the transdermal system, we prepared TDDSs with eight different polyacrylic resin II levels (C1–C8, Table 2) for the experiments and determined the 24 h cumulative release rate of CTH in vitro. The cumulative release rate of CTH in vitro gradually decreased with increasing concentrations of polyacrylic resin II. When no polyacrylic resin II was added, the transdermal CTH delivery system had the highest cumulative release rate of 68.13 ± 0.25% within 24 h (Figure 3). However, transdermal CTH delivery systems without polyacrylic resin II lack adhesion and stability. TDDSs with 1% and 2% polyacrylic resin II produced cumulative release rates of 59.91 ± 0.12 and 61.35 ± 0.77%, respectively, within 24 h, with no significant difference. Therefore, 2% was selected as the best level of polyacrylic resin II to ensure both a high level of CTH release and effective thickening and bonding.

### 2.5. Scanning Electron Microscopy of TDDS

**Figure 4:**
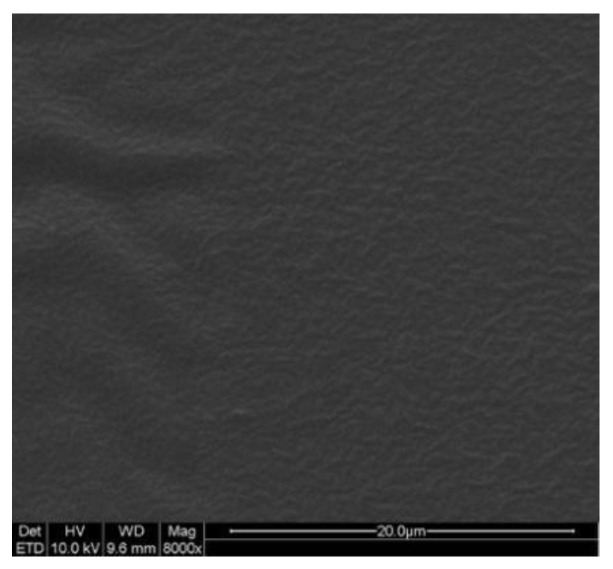
SEM image of the CTH transdermal drug delivery system. SEM: scanning electron microscopy. The images showed that the patch had good integrity and the drugs were evenly distributed in the polymer matrix.

Scanning electron microscopy (SEM) images of the CTH TDDS are shown in Figure 4. The images showed that the patch had good integrity and the drugs were evenly distributed in the polymer matrix, indicating that the prototype preparation had the necessary morphological characteristics for an effective TDDS.

### 2.6. Stability of the TDDS

Quality control experiments showed that at high or low temperatures and under freeze–thaw conditions, there was no flow, wrinkling, or embrittlement of the patch surface. Additionally, no oil leakage was observed on the back of the patches, and no delamination was observed between the patches and the lining. These results indicated that there was no significant change in the appearance or structure of the TDDS. Furthermore, these results indicated that the patches had good stability.

### 2.7. In Vivo Pharmacokinetic Studies of the TDDS

**Figure 5:**
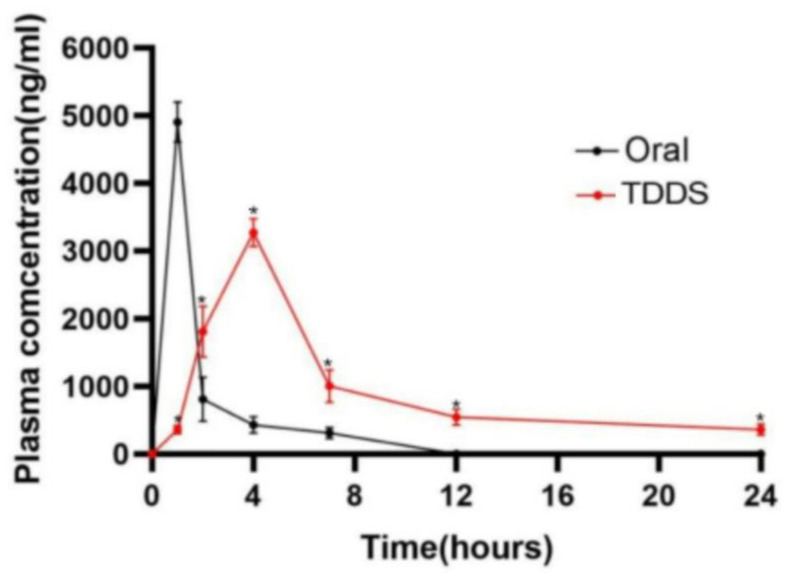
Plasma drug concentration–time curve analysis in rabbits after the administration of CTH via the transdermal drug delivery system and oral application. Data are presented as the mean ± SD (n = 6). * *p* < 0.05, vs. the oral administration group.

The pharmacokinetic performance of the optimized TDDS was compared to that of orally administered CTH in rabbits. The plasma concentration–time profiles are shown in Figure 5 and the relevant pharmacokinetic parameters are summarized in Table 3. Compared with oral administration, the patches took longer to reach the peak blood concentration, and the C_max_ was relatively lower. Furthermore, the AUC_(0-t)_ and MRT of the patches were significantly increased (*p* < 0.05), reaching 26,403.92 ± 2627.09 h‧ng/mL (4-fold) and 10.50 ± 0.83 h (1-fold), respectively. In addition, the ratio of the AUC_(0-t)_ for the TDDS and oral administration (a result called the calculated relative bioavailability or F value) was 312.09%. Therefore, the developed CTH TDDS exhibited significantly better sustained-release characteristics and higher bioavailability than oral administration.

### 2.8. Skin Irritation Tests

**Figure 6:**
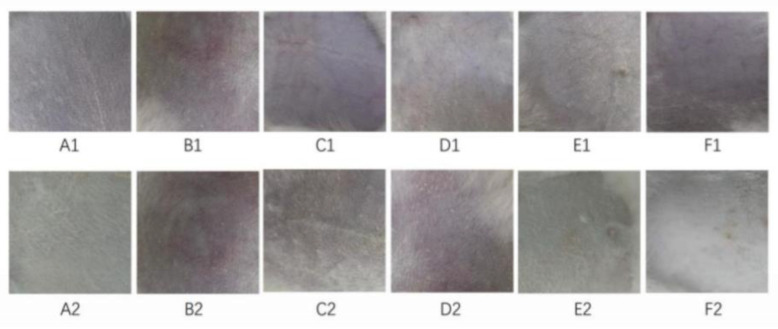
Skin irritation study of the CTH transdermal drug delivery system. (**A1**–**F1**): Before application of the patch; (**A2**–**F2**): after removal of the patch.

The results of the skin irritation tests following the use of CTH TDDS are shown in Figure 6. The patches did not induce erythema or edema in rabbit skin (a score 0.00–0.49 was considered non-irritating, Table 4). Furthermore, there was no significant difference in skin irritation between the blank TDDS and the CTH TDDS, and between 0 and 24 h after the CTH TDDS treatment (*p* < 0.05, Table 4). Therefore, the CTH TDDS is safe and does not irritate the skin.

### 2.9. Skin SEM after CTH TDDS Treatment

**Figure 7:**
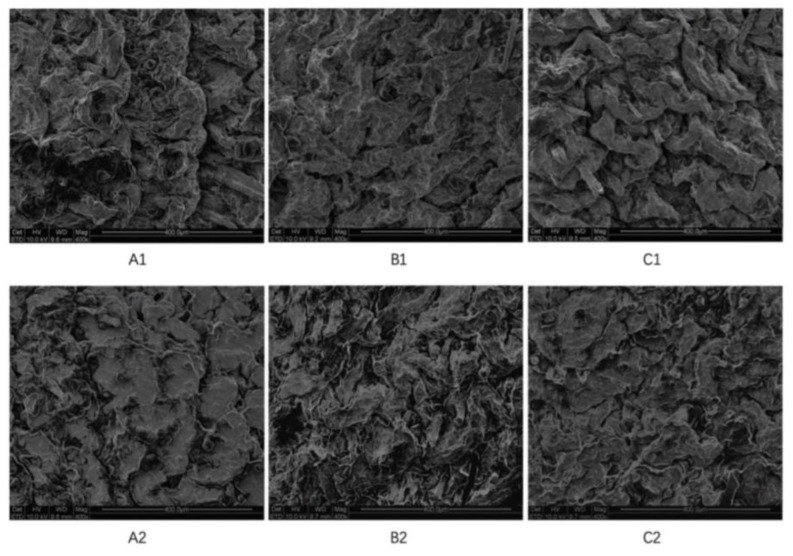
SEM images of the skin after transdermal administration of CTH. (**A1**–**C1**): Before application of the patch; (**A2**–**C2**): after removal of the patch. Images showed that the surface structure and morphology of the skin did not significantly change after the transdermal administration of CTH.

To further observe the microscopic appearance of the skin after the percutaneous delivery of CTH, we used SEM analysis of the mouse skin, as shown in Figure 7. It can be seen from the figure that the surface structure and morphology of the skin did not significantly change after the transdermal administration of CTH, and the skin shape was well maintained. 

## 3. Discussion

TDDSs can control drug release, which is beneficial for reducing side effects and improving the bioavailability of drugs [32]. TDDSs have two commonly used forms: a storage-type patch and a matrix-type patch, which seals the drug in a reservoir and provides better prescription flexibility [33,34]. Matrix-type patches disperse the drug in an adhesive or polymer matrix and coat it on an impermeable roof film to form a drug-containing layer, which regulates drug release by controlling the thickness and area of the drug-containing layer [35]. Matrix-based patches have many advantages over storage-based patches, such as a simple structure, low cost, ease of production, high safety, large loading capacity, and high bioavailability [36]. Therefore, a matrix-type patch was selected to prepare a transdermal CTH delivery system for this study.

The material used to construct matrix-based patches is very important. HPMC, EC, PVP K30, and PVA are four commonly used excipient materials with different characteristics [37,38,39,40]. HPMC is a hydrophilic slow-release excipient material with different viscosity grades (K4M, K15M, K30M, K100M, etc.) that can adjust the drug release rate by changing its viscosity [37]. ECs are nonionic polymers with low swelling, high gas permeability, and good mechanical strength and plasticity [38,39]. PVP K30 is a nonionic polymer with moisture absorption, adhesion, film formation, and dispersion properties [40]. PVA is a biocompatible and biodegradable polymer with high glass transition temperature, melting point, water swelling, and viscosity [41]. PVA combined with other polymers, such as HPMC or EC, can form an interpenetrating network structure and improve mechanical properties by providing film formation properties and drug release characteristics of the patch [42]. The results of the preliminary screening test in this study showed that an HPMCK_100M_:PVA ratio of 6:4 was the optimal composition of the scaffold material for the transdermal CTH drug delivery system.

In transdermal drug delivery systems, chemo-osmotic agents improve skin barrier function and promote the passage of drugs through the stratum corneum [43]. OA and azone are two commonly used chemo-osmotic agents. OA can increase lipid distribution and water content in the deep stratum corneum, whereas azone can cause uneven lipid distribution and reduced water content in the deep stratum corneum [44,45]. The combination of OA and azone improve the efficiency and stability of transdermal drug absorption [46]. The results of this study showed that 16% OA/azone (1:1) resulted in the highest cumulative release rate of CTH.

PSAs can bind to two objects under slight pressure at room temperature and are important components of a TDDS. Polyacrylic resin is a type of PSA, of which polyacrylic resin II is the most widely used [45]. Polyacrylic resin II can be used in TDDSs as an excipient layer, that is, a layer that does not contact the skin. Its role is to prevent the loss of active ingredients and water and to provide mechanical strength and flexibility [45]. In this study, different concentrations of polyacrylic resin II were added to detect and compare the cumulative release rates of CTH. The results showed that the concentration of polyacrylic resin II was 2%, which ensured a higher release rate and viscosity.

We evaluated the quality of the prepared CTH patch based on its optimal composition and examined its integrity, stability, and viscosity. First, we observed the morphological characteristics of the CTH patch using SEM, and the results showed that the appearance of the patch was complete, consistent, and flat [47,48]. Second, we stored the CTH patch under high-temperature, low-temperature, and freeze–thaw conditions, and the results showed no significant change under these conditions, indicating that its stability was good [41,42]. In addition, we evaluated the bonding properties of the CTH patches, including metrics such as peeling off, shear strength, and hand-felt viscosity [47,48]. Their results showed that the CTH patch achieved good adhesive performance. 

Effective blood concentrations of drugs can exert therapeutic effects. Some studies have reported that approximately 40–80 ng/mL citalopram hydrobromide can achieve a therapeutic effect [49,50]. In our study, the pharmacokinetics of the transdermal CTH delivery system were analyzed in rabbits. The results showed that the system had good absorption properties, showing a plasma CTH concentration above 0.05 μg/mL (effective drug concentration) within 24 h, prolonging the T_max_ to 4 h and the MRT (h) to 10.50 ± 0.83 h, indicating a good sustained-release effect. In addition, compared with oral gavage, the prepared transdermal system increased the AUC (0-t) value of CTH by more than three-fold, and the relative bioavailability of CTH was 312.09%, indicating significantly increased bioavailability. The existing study showed that between 40 ng/mL and 80 ng/mL of citalopram hydrobromide was an effective blood concentration in the common clinical dosage range (20 mg to 40 mg) [49,50]. In this study, the percutaneous delivery system of CTH was able to keep the plasma CTH concentration at 50 μg/mL within 24 h, within the estimated effective plasma concentration range, so it was considered that the patch could have a continuous therapeutic effect within 24 h.

Because the CTH patch is applied to the skin, an evaluation of whether it causes damage to the skin after application, such as erythema and edema, needed to be performed [51,52]. These reactions may be related to the drug components in the patch, the adhesives, or the solvents, as well as the retention time and frequency of the patch on the skin [51,52]. To evaluate the irritation caused by the TDDS, the skin was visually observed and analyzed using SEM and other techniques [51,52]. The skin of the rabbits used in these experiments was in good condition after the application of the CTH patch, with no erythema or edema, and there was no significant difference in the state of the skin in mice before and after administration, indicating that the CTH patch did not cause skin irritation.

## 4. Materials and Methods

### 4.1. Materials

Ethyl cellulose (EC; E809017, 18–22 mPa·s) was purchased from Shanghai Macklin Biochemical Technology Co., Ltd. (Shanghai, China). CTH (S48824), carboxymethyl cellulose sodium (S14016, 300–800 mPa·s), hydroxypropyl methylcellulose (HPMC, S14174, 4000–6500 mPa·s, HPMC K_4m_; 30,000 mPa·s, HPMC K_30m_; 100000 mPa·s, HPMC K_100m_), polyacrylic acid resin II (S30569), and hydroxyethyl cellulose (YY11927, 38,000–42,000 mPa·s) were purchased from Shanghai Yuan-ye Biotechnology Co., Ltd. (Shanghai, China). Potassium dihydrogen phosphate was purchased from Tianjin Huihang Chemical Technology Co., Ltd. (Tianjin, China). Azone, oleic acid (OA), polyvinylpyrrolidone (PVP K30, C20201, 3.4 mPa·s), and polyvinyl alcohol (PVA, D08019, 44.0–54.0 mPa·s) were purchased from Tianjin Damao Chemical Reagent Co., Ltd. (Tianjin, China). Methanol, ethanol, and acetonitrile were purchased from Tianjin Concord Technology Co., Ltd. (Tianjin, China). Methanol and acetonitrile were of high-performance liquid chromatography (HPLC) grade, while other reagents were of analytical grade.

### 4.2. Animals

Female Kunming mice (6 weeks old, weighing 16–18 g) and female Chinese rabbits (6 months old, weighing 2.0 kg) were purchased from Beijing SPF Biotechnology Co., Ltd. (Beijing, China). The mice were housed under standard laboratory conditions (12 h light/dark cycle, ~25 °C) with ad libitum access to food and water. Animals were housed in individual cages and fed a standard diet. Animal care and experimental protocols followed the Institutional Guidelines for the Health and Care of Experimental Animals. The study protocol was approved by the Committee for Ethics of Animal Experiments at Nankai University, Tianjin, China.

### 4.3. Drug Determination

CTH in the samples was quantified using a CoM 6000 HPLC system (CoMetro Technology, South Plainfield, NJ, USA) with a Comatex C18-AB column (250 × 4.6 mm, 5 μm; 115 Corp, Plainfield, NJ, USA) at 238 nm. The mobile phase was 0.1 mol/L acetonitrile, 0.1 mol/L ammonium acetate, and 0.01 mol/L heptane sodium sulfonate at a ratio of 5:5:1 (*v*/*v*). The flow rate was 1.0 mL/min, and the column temperature was 25 °C. All sample solutions were degassed and the injection volume was maintained at 20 μL [44,53]. The particular application detail on the CoM 6000 HPLC system referred to a previous introduction in our laboratory [30,31].

### 4.4. Preparation of Mouse Abdominal Skin

Cervical dislocation was performed in Kunming mice under 4% chloral hydrate anesthesia. Hair was gently removed from the mice using a hair removal device (a sharp razor), and a skin sample (approximately 2 × 2 cm) with no damage was cut from the abdominal region. After removing the subcutaneous adipose tissue and adhesive layer, the skin sample was repeatedly washed with normal saline and stored at −20 °C in a Petri dish covered with filter paper wetted with normal saline. Skin samples were thawed at 25 °C before use.

### 4.5. Optimization of Transdermal Systems

Components of the CTH transdermal delivery system were screened using in vitro transdermal release assays. The compositions of the three categories of formulations of primary and secondary excipient materials and osmotic agents are shown in Table 5. The basic composition of the CTH patch was determined by investigating the effect of each component on the drug release rate through a preliminary screening test. Next, a single-factor test was performed to optimize the ratio of the different formulations including excipient material, penetration enhancer, and resin to determine the best formula (Table 2). Firstly, the 9 different excipient material ratios were be tested based on Table 2, A1–A9. Secondly, the 9 different penetration enhancer concentrations were be tested based on Table 2, B1–B9. Finally, the 8 different polyacrylic resin II concentrations were tested based on Table 2, C1–C8.

### 4.6. In Vitro Release Studies

Drug-release studies were conducted using a Franz transdermal diffusion meter (TP-6 transdermal diffusion meter; Tianjin Jingtuo Instrument Technology Co., Ltd., Tianjin, China) referring to the methods used many times in our laboratory, and a TP-6 transdermal diffusion meter was introduced in detail [30,31]. The effective diffusion area of the Franz transdermal diffusion cell is 3.14 cm^2^ [30,31]. The excised skin was flattened and firmly affixed between the donor and receptor compartments. Each receptor compartment was filled with 15 mL of saline solution, maintained at 37 ± 0.5 °C using a thermostat, and stirred with a magnetic bar at 70× *g* to maintain a uniform concentration. A 0.5 mL sample was taken from the recipient compartment 24 h after transdermal delivery began. The samples were filtered using a 0.22 μm pinhole filter (Scienhome Scientific Technology Development Co., Ltd., Tianjin, China) before injection into the HPLC instrument.

### 4.7. Preparation of the TDDS

Drug-containing transdermal systems were prepared using a solvent evaporation technique, according to the ratios described in Table 6, with 20 mg CTH. Each component was dissolved in 75% ethanol and magnetically stirred (60 °C, 30× *g*) for 2 h. After the solution was evenly mixed, it was degassed via ultrasonication at room temperature for 30 min. Next, the mixture was spread on an anti-adhesion layer and dried at 45 °C for 15 min, and subsequently pressed onto the dialysis membrane surface to obtain the transdermal systems. The patches were stored at room temperature in sealed containers.

### 4.8. Scanning Electron Microscopy Analysis

The external morphology of the TDDS was analyzed using a scanning electron microscope (QUANTA 200; FEI Company, Hillsboro, OR, USA).

### 4.9. Quality Determination of the Prepared TDDS

#### 4.9.1. High-Temperature Experiment

The CTH TDDSs were placed in a heating chamber at 50 ± 5 °C for 7 days. The exterior appearances and spreading properties of the patches were examined. All experiments were performed in triplicate.

#### 4.9.2. Cold Resistance Experiment

The CTH TDDSs were placed in a refrigerator at 2–8 °C for 7 days. This process was repeated three times, and the exterior appearance and ductility of the patches were examined at room temperature.

#### 4.9.3. Freeze–Thaw Experiment

The CTH TDDSs were placed alternately in a freezer at −20 °C and an oven at 37 ± 0.5 °C for 8 days. This process was performed in triplicate and the exterior of each patch was examined as described above.

### 4.10. In Vivo Pharmacokinetic Studies

The rabbits were assigned to one of the following three groups: A, B, or C. The abdominal hair of the rabbits in groups B and C was shaved off with an electric hair remover, and after cleaning the skin with normal saline, the optimized TDDS was applied to the abdomens of mice in group B, and blank patches were applied to the abdomens of rabbits in group C. Group A was administered CTH by oral gavage. The oral gavage procedure involved fixing the rabbits with an animal fixator and placing a special mouth expander with a rope attached to their mouth. An elastic rubber catheter was inserted through a small round hole in the mouth expander and pushed into the esophagus along the posterior pharyngeal wall. A solution consistent with the TDDS concentration was added. After gastric perfusion, the catheter and mouth opener were removed. Next, 1 mL aliquots of blood were collected from the marginal auricular vein of the experimental animals at different time intervals (1, 2, 4, 7, 12, and 24 h), placed in heparinized tubes, and centrifuged at 1500× *g* for 10 min. The resulting plasma supernatant was stored at −70 °C until further analysis. HPLC analyses were carried out by transferring 200 μL of the supernatant to a 1.5 mL polyethylene centrifuge tube. The tubes were then mixed with 300 μL acetonitrile, centrifuged at 2500× *g* for 10 min, and filtered through a 0.22 μm membrane.

### 4.11. Skin Irritation Studies

After the patch treatment, the test substances were removed simultaneously from groups B and C. Erythema and edema in the depilated part of the abdomen in the two rabbit groups were observed and recorded at 0 and 24 h after removing the test substance. Erythema and edema (both level 0–4, and score 0.00–0.49 was considered non-irritating) at the application site were also evaluated.

### 4.12. Data Analysis

The pharmacokinetic parameters of the conventional oral and sustained-release transdermal preparations were analyzed, including the maximum plasma concentration of the drug (C_max_), the time to reach the C_max_ (T_max_), the mean residence time (MRT), and the area under the plasma concentration–time curve from time 0 to t (AUC_(0-t)_). Two-tailed Student’s *t*-tests were performed for the C_max_ and AUC_(0-t)_ analyses, and a non-parametric test was performed for the T_max_ analysis. The AUC_(0-t)_ was calculated using the trapezoidal rule.

All results are represented as the mean ± standard deviation, and comparisons were performed using one-way analysis of variance. Statistical significance was set at *p* < 0.05.

## 5. Conclusions

Based on both in vitro and in vivo data, we prepared and optimized a CTH TDDS as a patch formulation consisting of 49.2% HPMCK_100M_, 32.8% PVP K30, 16% OA:azone (1:1), and 2% polyacrylic resin II. The patch prepared with this formulation exhibited satisfactory integrity, stability, and viscosity, and induced no irritation after ideal skin application. In addition, the patch slowly released CTH, thereby prolonging its antidepressant effects. However, further clinical studies are required to fully evaluate the prospects of this TDDS.

## Figures and Tables

**Table 1:** Preliminary screening of formulations.

	First Excipient Materials	Second Excipient Materials	Penetration Enhancers	Cumulative Release Rate (%)
1	1	1	1	0.25 ± 0.08 ^a^
2	1	1	2	14.79 ± 0.51 ^b^
3	1	1	3	26.42 ± 0.84 ^c^
4	1	2	1	6.75 ± 0.82 ^d^
5	1	2	2	16.49 ± 0.80 ^e^
6	1	2	3	3.17 ± 0.42 ^f^
7	1	3	1	10.26 ± 0.84 ^g^
8	1	3	2	20.23 ± 0.59 ^h^
9	1	3	3	2.89 ± 0.84 ^i^
10	2	1	1	4.10 ±0.68 ^j^
11	2	1	2	26.57 ± 0.99 ^c^
12	2	1	3	14.85 ± 0.87 ^b^
13	2	2	1	2.23 ± 0.96 ^fik^
14	2	2	2	1.92 ± 0.40 ^k^
15	2	2	3	5.86 ± 0.54 ^l^
16	2	3	1	6.60 ±0.68 ^d^
17	2	3	2	7.70 ±0.40 ^m^
18	2	3	3	8.57 ± 0.50 ^n^
19	3	1	1	3.57 ± 0.93 ^fj^
20	3	1	2	22.14 ± 0.64 ^o^
21	3	1	3	40.82 ± 0.86 ^p^
22	3	2	1	0.21 ± 0.08 ^a^
23	3	2	2	6.74 ± 0.39 ^d^
24	3	2	3	0.21 ± 0.04 ^a^
25	3	3	1	0.86 ± 0.71 ^a^
26	3	3	2	8.96 ± 0.32 ^n^
27	3	3	3	20.19 ± 0.26 ^h^

Notes: the same letters mean *p* > 0.05, and the different letters mean *p* < 0.05; the following figures and tables are the same.

**Table 2:** Optimization of the CTH transdermal drug delivery system.

Formulation	HPMCK_100M_:PVA	OA:Azone (1:1) (%, *v/v*)	Polyacrylic Acid Resin II (%, *v/v*)
A1	1:9		
A2	2:8		
A3	3:7		
A4	4:6		
A5	5:5		
A6	6:4		
A7	7:3		
A8	8:2		
A9	9:1		
B1		0	
B2		4	
B3		8	
B4		13	
B5		16	
B6		20	
B7		24	
B8		28	
B9		32	
C1			0
C2			1
C3			2
C4			3
C5			4
C6			5
C7			6
C8			8

**Table 3:** Pharmacokinetic parameters (n = 6).

Parameters	Oral Administration	LP-VPH Compound Patch
C_max_ (ng/mL)	4902.10 ± 395.69	3269.84 ± 207.45 *
T_max_ (h)	1	4 *
AUC_(0-t)_ (ng·h^−1^·mL^−1^)	8460.37 ± 996.43	26,403.92 ± 2627.09 *
MRT (h)	2.40 ± 0.25	10.50 ± 0.83 *
F (%)	-	312.09

Notes: * *p* < 0.05, vs. oral group.

**Table 4:** Scores of skin erythema and edema in rabbits treated with patches (n = 6).

Administration	Erythema		Edema	
	0 h	24 h	0 h	24 h
Blank patch	0.00 ± 0.00	0.34 ± 0.07	0.00 ± 0.00	0.14 ± 0.06
CTH patch	0.00 ± 0.00	0.45 ± 0.16	0.00 ± 0.00	0.20 ± 0.07

**Table 5:** Alternative components of the CTH transdermal delivery system.

Serial Number	First Excipient Materials	Second Excipient Materials	Penetration Enhancers
1	HPMCK_4M_	PVA	OA
2	HPMCK_30M_	PVP K30	AZONE
3	HPMCK_100M_	EC	OA:AZONE (1:1)

**Table 6:** CTH transdermal drug delivery system optimized formulation.

Composition	Proportion (%)
HPMCK_100M_	49.2% (*w*/*w*)
PVA	32.8% (*w*/*w*)
OA:azone = 1:1	16% (*v*/*v*)
Polyacrylic Acid Resin II	2% (*w*/*w*)

## Data Availability

Data are contained within the article.
